# Implications of analysing time-to-event outcomes as binary in meta-analysis: empirical evidence from the Cochrane Database of Systematic Reviews

**DOI:** 10.1186/s12874-022-01541-9

**Published:** 2022-03-20

**Authors:** Theodosia Salika, Rebecca M. Turner, David Fisher, Jayne F. Tierney, Ian R. White

**Affiliations:** MRC Clinical Trials Unit, Institute of Clinical Trials and Methodology, University College London, London, UK

**Keywords:** Time-to-event, Meta-analysis, Methodology, Survival data, Clinical trials, Cochrane database of systematic reviews

## Abstract

**Background:**

Systematic reviews and meta-analysis of time-to-event outcomes are frequently published within the Cochrane Database of Systematic Reviews (CDSR). However, these outcomes are handled differently across meta-analyses. They can be analysed on the hazard ratio (HR) scale or can be dichotomized and analysed as binary outcomes using effect measures such as odds ratios (OR) or risk ratios (RR). We investigated the impact of reanalysing meta-analyses from the CDSR that used these different effect measures.

**Methods:**

We extracted two types of meta-analysis data from the CDSR: either recorded in a binary form only (“binary”), or in binary form together with observed minus expected and variance statistics (“OEV”). We explored how results for time-to-event outcomes originally analysed as “binary” change when analysed using the complementary log–log (clog-log) link on a HR scale. For the data originally analysed as HRs (“OEV”), we compared these results to analysing them as binary on a HR scale using the clog-log link or using a logit link on an OR scale.

**Results:**

The pooled HR estimates were closer to 1 than the OR estimates in the majority of meta-analyses. Important differences in between-study heterogeneity between the HR and OR analyses were also observed. These changes led to discrepant conclusions between the OR and HR scales in some meta-analyses. Situations under which the clog-log link performed better than logit link and vice versa were apparent, indicating that the correct choice of the method does matter. Differences between scales arise mainly when event probability is high and may occur via differences in between-study heterogeneity or via increased within-study standard error in the OR relative to the HR analyses.

**Conclusions:**

We identified that dichotomising time-to-event outcomes may be adequate for low event probabilities but not for high event probabilities. In meta-analyses where only binary data are available, the complementary log–log link may be a useful alternative when analysing time-to-event outcomes as binary, however the exact conditions need further exploration. These findings provide guidance on the appropriate methodology that should be used when conducting such meta-analyses.

**Supplementary Information:**

The online version contains supplementary material available at 10.1186/s12874-022-01541-9.

## Background

Systematic reviews and meta-analyses of time-to-event outcomes (e.g. time to death, recurrence of symptoms, relief of pain etc.) are frequently carried out in areas such as cancer, respiratory and cardiovascular diseases, since event timings are crucial to assessing the impact of an intervention [1]. The decision on how time-to-event outcomes are handled in a particular meta-analysis largely depends on how eligible studies are reported, and is usually out of the control of the meta-analyst except if individual participant data (IPD) are available. The information extracted by systematic reviewers may include the total number of participants and events per arm, and/or the hazard ratio alongside its confidence interval, and/or the log-rank observed minus expected statistic (“O-E”) and its variance (“V”) (which are useful alternative statistics if a hazard ratio is not directly reported [1]). Time-to-event data can be analysed using the effect measure of hazard ratio (HR), or can be dichotomised and analysed as binary using effect measures such as the odds ratio (OR) or risk ratio (RR) [2]. Although HR is considered the most appropriate scale for analysis of time-to-event data, in practice OR and RR are frequently used instead due to the following reasons: unavailability of individual participant data (IPD); limitations on how these outcomes are reported in individual trial reports; lack of familiarity in handling time-to-event outcomes for meta-analysis; difficulties in understanding the methods of analysing such data without a statistician; limited available training for the majority of systematic reviewers and meta-analysts who perform such analyses [3].

In the past, research was conducted comparing the differences between the OR using logistic regression models and the HR using proportional hazard (PH) models within individual studies. Green and Symons [4] showed that logistic and Cox PH models produce similar results when the event is rare and for shorter follow-up times under a constant hazard rate. Ingram and Kleinman [5] added that important differences among the methods occur in the presence of varying censoring rates and length of follow-up. However, it has not been established yet how such results transfer to the context of an aggregate data meta-analysis for which summary data is extracted from trial reports. Further, in this context it is of interest to examine potential alternatives such as the use of the complementary log–log link, which may reduce the difference in the results between the two effect measures used. The overall meta-analytic estimate can be affected due to changes to the weighting allocated to each study, and therefore changes to the results can be unpredictable. We aimed to carry out an empirical “meta-epidemiological” study using survival meta-analysis data from the Cochrane Database of Systematic Reviews (CDSR) (Issue 1, 2008) to explore the implications of analysing time-to-event outcomes as binary in meta-analysis. We assessed the importance of extracting suitable data such as the “O-E” and “V” statistics rather than binary summaries to perform such analyses; in the occasion where binary data were available we examined whether the use of alternative methodology such as the complementary log–log link (clog-log), proven to facilitate interpretation of the results on a HR scale [6, 7] can minimise the error we may observe in the results. We assess only the differences between the OR and the HR, as the RR, according to the literature [8–11], is placed in between these measures and therefore, we expect to capture any bias within these extremes. We perform these analyses under both two- and one-stage models.

The rest of the paper is set out as follows. In the methods section, we describe the dataset we used and the statistical models that we applied. In the results, we present descriptive statistics of the database and then we describe the results obtained from reanalysing the data originally analysed as binary on an HR scale and from reanalysing the data originally analysed using “O-E” and “V” data on an OR scale. These results are followed by a discussion exploring the strengths and limitations of our findings, together with conclusions and implications.

## Methods

### Data

The Nordic Cochrane Centre provided the content of the first issue from 2008 of the CDSR. The database includes meta-analyses within reviews which have been classified previously by outcome type, medical specialty and types of interventions included in the pairwise comparisons [12]. The database did not record whether data type was time-to-event; however, based on the outcome classification we were able to identify (using words such as “survival”, “death”, “fatality”) three sets of time-to-event meta-analyses:

- “binary”: Those with outcome classification “all-cause mortality” where the information recorded was based only on the number of events and participants per arm;
- “OEV”: Those with outcome classifications “overall survival” and “progression/disease free survival” where the information recorded was based on “binary” data in addition to log-rank “O-E” and “V” statistics”; these were originally analysed as HRs in the RevMan software;
- Those with estimated log HR and its standard error. These were removed from further analyses since there was no available information on the number of events and participants per arm and therefore no binary data meta-analysis could be conducted.

Therefore, we identified two subsets of time-to-event meta-analyses: those with binary summaries, and those with binary summaries in addition to OEV data; we analysed each outcome per dataset separately to assess whether differences exist due to different characteristics of the outcomes. We also examined whether the information obtained from “OEV” data was based on aggregate data or IPD by examining the individual Cochrane reviews.

### Eligibility Criteria

**Fig. 1:**
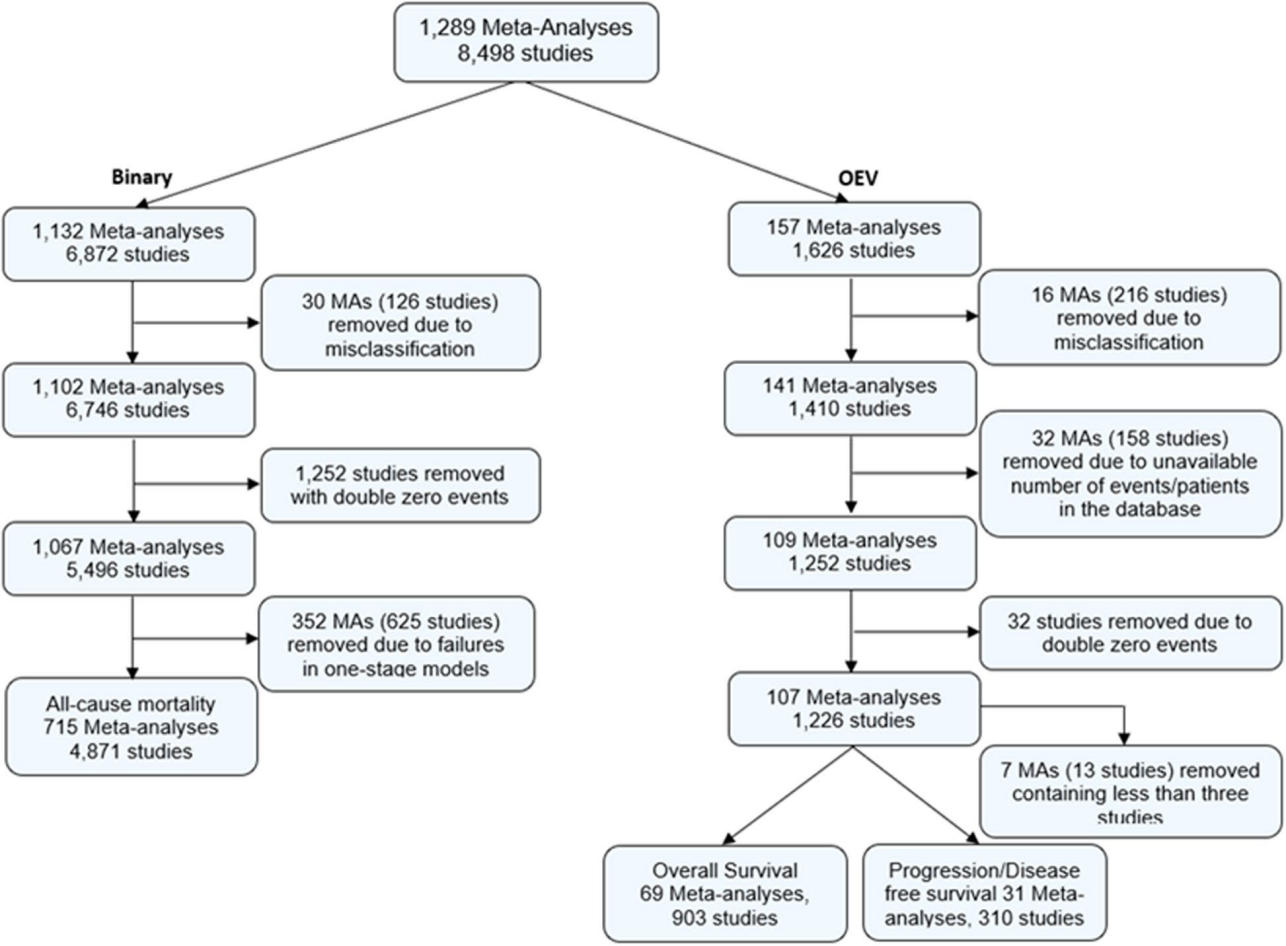
Analysis sample of “binary” and “OEV” datasets from the CDSR (2008, issue 1)

RMT (for “binary” data) and TS (for “OEV” data) initially extracted these data and conducted cleaning including examination of the outcome classification; TS repeated the “binary” data extraction to confirm the information obtained were accurate and RMT confirmed the choice of included meta-analyses obtained from “OEV” data extraction. Both datasets could contribute more than one meta-analysis per Cochrane review. RMT and TS identified 46 misclassifications due to disagreement with the original outcome classification as listed in the datasets, conflicting information in the database or unavailability of the correct version of the Cochrane review. We excluded 1,284 studies including double zero events, since they do not contribute to the meta-analysis results [12, 13]. We removed another 359 meta-analyses including fewer than 3 studies because some of the models applied below (i.e. generalised linear mixed models) will be affected by estimation issues and inevitable failures using small numbers of studies [14]; hence we wanted to make fair comparisons between the models applied. Derivation of the analysis sample is provided in Fig. 1.

### Descriptive statistics

We describe the number of studies per meta-analysis, number of events and study size by the median and interquartile range. We also identify the number of medical specialities, and median number of events (and interquartile range) per medical specialty.

### Model description for “binary” data

We used the following meta-analysis models to analyse the data on the OR or HR scale. The first was a model proposed for “binary” data (assuming a binomial likelihood with a logit link) which is based only on the number of patients and number of events which occurred. Interpretation for the treatment effect is conducted in terms of the logarithm of an OR.

In the second approach, we modelled the binary data using a normal approximation to binomial likelihood with a complementary log–log link (clog-log), where treatment effect interpretation was based on the logarithm of a HR. This method is also based only on the number of patients and events which occurred, and ignores censoring and the time element; however it is closely related to continuous-time models, has a built-in proportional hazards assumption, and therefore has important application in survival analysis [6].

### Fitting two-stage random-effects models for “binary” data

Prior to fitting the two-stage random-effects models, study arms with zero events were identified for the “binary” data. For 771 studies, a “treatment arm” continuity correction was applied as proposed by Sweeting et al. [15] and was constrained to sum to one as this ensures that the same amount of information is added to each study.

Let $i=\mathrm{1,2},\dots ,n$ denote the study. The estimated log odds and log hazard ratios were given by:

$$y_i=\left\{\begin{array}{lll}\log{\left(\frac{{\mathrm A}_{\mathrm i}}{{\mathrm B}_{\mathrm i}}\right)}-\log{\left(\frac{{\mathrm C}_{\mathrm i}}{{\mathrm D}_{\mathrm i}}\right)} \mathrm {for}\ \mathrm{ORs} & \qquad(1) \\{\log}{\left[-\log{\left(1-P_{Ti}\right)}\right]}-\log{\left[-\log{\left(1-P_{Ci}\right)}\right]}\mathrm {for}\ \mathrm {HRs} & \qquad(2)\end{array}\right.$$

where ${\mathrm{A}}_{\mathrm{i}},{\mathrm{C}}_{\mathrm{i}}$ represented number of events, ${\mathrm{B}}_{\mathrm{i}},{\mathrm{D}}_{\mathrm{i}}$ represented number of non-events in the treatment and control groups respectively, ${P}_{Ti}=\frac{{\mathrm{A}}_{\mathrm{i}}}{{\mathrm{A}}_{\mathrm{i}}+{\mathrm{B}}_{\mathrm{i}}}$ was the proportion of events on the treatment arm of the ${i}^{th}$ study, and ${P}_{Ci}=\frac{{\mathrm{C}}_{\mathrm{i}}}{{\mathrm{C}}_{\mathrm{i}}+{\mathrm{D}}_{\mathrm{i}}}$ was the proportion of events on the control arm of the ${i}^{th}$ study.

The corresponding variances were given by:

$$s_i^2\;=\;\left\{\begin{array}{lll}\frac1{A_i}\;+\;\frac1{B_i}\;+\;\frac1{C_i}\;+\;\frac1{D_i}\;for\;ORs & \qquad(3) \\ \left(\frac1{\log\;\left(1-P_{Ti}\right)\ast\left(P_{Ti}-1\right)}\right)^2\;\ast\;\left(\frac{P_{Ti\ast}\left(1-P_{Ti}\right)}{A_{\mathrm i}\;+\;B_{\mathrm i}}\right)\;+\;\left(\frac1{\log\left(1-P_{Ci}\right)\ast\left(P_{Ci}\;-\;1\right)}\right)^2\;\ast\;\left(\frac{P_{Ci}\ast\left(1-P_{Ci}\right)}{{\text{C}}_\text{i}\;+\;{\text{D}}_\text{i}}\right)\;for\;HRs & \qquad(4)\end{array}\right.$$

Equations 2 and 4 provided a HR estimate via the use of the complementary log–log link considered as a useful link function for the discrete-time hazards models as recommended by Hedeker et al. [7] and Singer et al. [6]. We estimated the study-specific log odds ratios or log hazard ratios, ${y}_{i}$ and their within-study variances ${s}_{i}^{2}$ as shown above and fitted a standard two-stage random-effects model to these. Additionally, we obtained the ${I}^{2}$ statistic from the fitted models as follows:

$${I}^{2}=\frac{{\widehat{\tau }}^{2}}{{\widehat{\tau }}^{2}+{\widehat{\sigma }}^{2}}$$

where ${\tau }^{2}$ denotes the variance of the underlying true effects across studies and ${\sigma }^{2}$ the typical within-study variance.

To avoid downward bias in the variance components estimates, we used the REML estimator for model implementation [16]. The models were implemented via the “rma.uni” command from “metafor” package in R. We also fitted one-stage random-effects models for “binary” data. The methods related to one-stage meta-analysis models and code is available in Additional file 1.

### Model description for “OEV” data

For “OEV” data, the “O-E” and “V” statistics were available in the Cochrane database alongside the number of patients and events. These data came either from published reports or from IPD; TS examined the individual reviews from the Cochrane database and assessed the data origin. Since there were more available information for these data the following three models were applied, using only two-stage meta-analysis models.

Similarly to “binary” data, we initially analysed the “OEV” data as “binary” and modelled them as described in detail in the preceding section. We also used the log-rank Observed—Expected events (O-E) and the log-rank Variance (V) statistics calculated previously from the number of events and the individual times to event on each research arm of the trial; we used the log-rank approach [17] in order to obtain another type of HR estimate. We used random-effects models to analyse the data throughout, including between-study heterogeneity to account for variation across studies.

### Fitting two-stage random-effects models for “OEV” data

Similarly to the “binary” data, the estimated log odds and log hazard ratios were given by Eqs. 1 and 2 for the binary summaries while the “O-E” and “V” statistics were used as follows:

5 $$y_i=\frac{logrank\ Observed-Expected\ events\ (O-E)}{logrank\ Variance\ (V)}\ for\ HRs$$

The corresponding variances were given by Eqs. 3 and 4 for binary summaries while for “O-E” and “V” statistics as follows:

6 $$s_i^2=\frac{1}{logrank\ Variance\ (V)}\ for\ HRs$$

where $V$ denotes the variance of the logrank statistic. We used the REML estimator for model implementation [16] and the models were implemented via the “rma.uni” command from “metafor” package in R.

### Model comparison for “binary” data

The following model comparisons were performed. For the “binary” data set, we examined whether the results from analysing survival data as binary on an OR scale are similar to results from analysing on the HR scale using the clog-log link, both under two-stage and one-stage models. For presentation purposes, we present only comparisons of the results under two-stage models in the main paper (and for one-stage models in the Additional file 1) in order to assess the discrepancies between the model using the logit link and the model using the complementary log–log link.

First, we examined the proportion of significant and non-significant meta-analytic pooled effect estimates under the different scales used (OR vs HR scale); we identified the number of meta-analyses which were significant under one scale and non-significant under the other at a two-sided 5% level of significance.

Bland–Altman plots with associated 95% limits of agreement were constructed, with the aim of facilitating interpretation of results and producing fair comparisons between the two scales [18]. In order to create these plots, results were standardised by dividing the logarithm of the estimate by its standard error. Plots were produced for the standardised treatment effect estimates and for the ${I}^{2}$ statistics. ${I}^{2}$ represents the percentage of variability that is due to between-study heterogeneity rather than chance; ${I}^{2}$ values range from 0 to 100%. This measure was chosen for model comparison as it enables us to compare results directly between the two scales used. The variance of underlying true effects across studies (${\tau }^{2}$) was not used as it does not allow direct comparison between different outcome measures.

We identified “outliers” as meta-analyses outside the 95% limits of agreement, and we examined their characteristics. The meta-analysis characteristics we examined were the following:

- between-scale differences in the magnitude of the pooled treatment effect estimate and its 95% confidence intervals
- the levels of within-study standard error and between-study heterogeneity and study weights in the meta-analysis
- study-specific event probabilities and baseline risk

We summarised these differences by meta-analysis and reported those characteristics which were mostly associated with substantial differences between OR pooled effect estimates and corresponding HR pooled effect estimates.

### Model comparison for “OEV” data

For the “OEV” data set, comparisons on overall and progression disease free survival outcomes were conducted separately; this was because differences between these outcomes might be observed in the presence of different disease severities, and therefore this would be associated with different length of follow-up and risk of the outcome.

For both outcomes, we performed comparisons by examining the differences between analysing the data as binary on an OR scale, analysing the data as binary using the clog-log link on a HR scale, or analysing the data using the “O-E” and “V” statistics on a HR scale. We assessed whether the differences observed from analysing the data as binary on an OR scale could be reduced by the use of the clog-log link. We present only comparisons of the results under two-stage models since there were no available IPD to perform comparisons under one-stage models.

Similarly to “binary” data, we examined the proportion of significant and non-significant meta-analytic pooled effect estimates under the different scales used and identified the number of meta-analyses which were significant under one scale and non-significant under the other. We created Bland–Altman plots for the standardised treatment effect estimates and for the ${I}^{2}$ statistics to explore the agreement among the methods producing fair comparisons between the two scales [18]. Meta-analyses outside the 95% limits of agreement were examined for their characteristics.

## Results

### Results for “binary” data

For the outcome of “all-cause mortality”, 1,132 meta-analyses within the Cochrane database were originally analysed as binary. The median number of meta-analyses per review was 1 with IQR (1,2). The median number of studies and the median number of events are provided in Table 1, indicating that these numbers were a lot smaller than those obtained for the “OEV” data.

**Table 1:** Descriptive statistics for “binary” and “OEV” data from the CDSR

	*“binary”*	
**Outcome**	**All-cause Mortality**	
Total Number of MA	715	
Number of studies per MA: Median (IQR)	5 (3, 8)	
Number of events per MA: Median (IQR)	13 (4, 40)	
Median Study Size (IQR)	124 (60, 312)	
	*“OEV”*	
**Outcome**	**Overall Survival**	**Progression/Disease Free Survival**
Total Number of MA	69	31
Number of studies per MA: Median (IQR)	10 (6, 14)	10 (7, 14)
Number of events per MA: Median (IQR)	108 (58, 254)	104 (70, 192)
Median Study Size (IQR)	182 (93, 369)	185 (90, 317)

The distribution of medical specialities of the meta-analyses is presented in Table 2. For the “binary” data, “Cardiovascular” (23%) is the most frequently occurring category, followed by “Cancer” (13%), “Gynaecology, pregnancy and birth” (12%) and “respiratory diseases” (12%). The median number of events in cancer substantially exceeded the median number of events in other medical areas.

**Table 2:** Distribution of medical specialties for the “binary” and “OEV” data meta-analyses in the CDSR

	***“binary”***			
**Medical Specialty**	**ACM**^**b**^ **Number (%) of MAs**	**Events per MA:** **Median (IQR)**		
Cancer	95 (13%)	49 (17, 120)		
Cardiovascular	168 (23%)	14 (4, 43)		
Central nervous system/musculoskeletal	44 (6%)	12 (5, 33)		
Digestive/endocrine, nutritional and metabolic	71 (10%)	7 (3, 18)		
Gynaecology, pregnancy and birth	87 (12%)	7 (2, 20)		
Infectious diseases	46 (6%)	18 (8, 47)		
Mental health and behavioural conditions	21 (3%)	2 (1, 5)		
Pathological conditions, symptoms and signs	5 (1%)	9 (2, 15)		
Respiratory diseases	87 (12%)	11 (5, 36)		
Urogenital	30 (4%)	4 (2, 12)		
Other^a^	61 (9%)	9 (3, 27)		
	***“OEV”***			
**Medical Specialty**	**OS**^**c**^**:** **Number (%) of MAs**	**Events per MA: Median (IQR)**	**PDFS**^**d**^**: Number (%) of MAs**	**Events per MA: Median (IQR)**
Cancer	60 (87%)	104 (45, 221)	31 (100%)	116 (56, 243)
Digestive/endocrine, nutritional and metabolic	1 (1%)	52 (35, 64)	-	-
Infectious diseases	8 (12%)	482 (160, 1109)	-	-

^a^Other: Blood and immune system, General heath, Injuries, Mouth and dental, and Cystic fibrosis

^b^*ACM* All-cause mortality;

^c^*OS* Overall Survival;

^d^PDFS: Progression/Disease free survival

Once the models were applied, we compared results between OR and HR analyses. Table 3 provides the percentages of significant and non-significant meta-analyses at a two-sided 5% level of significance indicating that there are few discrepancies present for both “binary” and “OEV” datasets under two-stage models.

**Table 3:** Number (%) of (non-)significant meta-analyses under different scales for two-stage models (“binary” and “OEV” data)

	**Outcome**		**OR**		**HR (O-E & V)**	
***“binary”***						
			Significant	Non-significant	Significant	Non-Significant
**HR** **(clog-log)**	All-cause mortality	Significant	106 (15%)	2 (0.1%)		
		Non-significant	4 (0.6%)	603 (84%)		
***“OEV”***						
			Significant	Non-significant	Significant	Non-Significant
**HR** **(clog-log)**	Overall Survival	Significant	20 (29%)	1 (0.2%)	18 (26%)	10 (14%)
		Non-significant	1 (0.2%)	47 (68%)	3 (4%)	38 (55%)
	Progression / Disease free Survival	Significant	9 (29%)	0 (0%)	8 (26%)	6 (19%)
		Non-significant	1 (3%)	21 (68%)	1 (3%)	16 (52%)
**HR** **(O-E &V)**	Overall Survival	Significant	18 (26%)	10 (14%)		
		Non-significant	3 (4%)	38 (55%)		
	Progression / Disease free Survival	Significant	9 (29%)	5 (16%)		
		Non-significant	1 (3%)	16 (52%)		

**Fig. 2:**
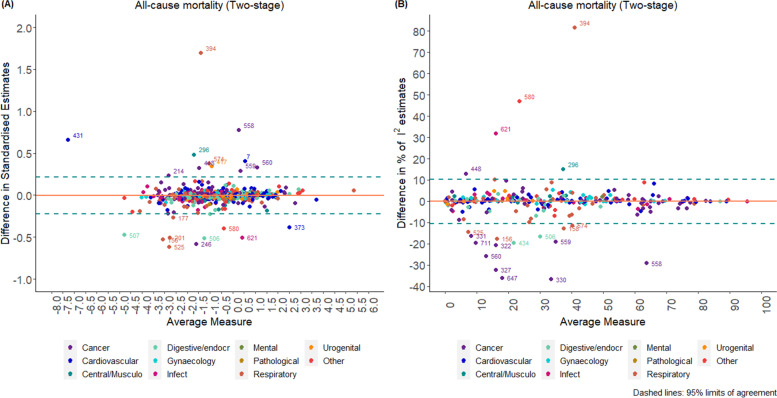
Bland–Altman plots comparing standardised pooled effect and ${I}^{2}$ estimates for two-stage models (“binary” data)

According to the Bland–Altman plot (Fig. 2), the average difference between the two methods for the standardised pooled effect estimates was -0.004 units (-0.222 units, 0.214 units) and -0.1% (-10.6%, 10.3%) for the estimation of I^2^ for two-stage models; this indicates a relatively small percentage difference between the two methods in the estimation of the measure of impact of heterogeneity I^2^. The width of the 95% limits of agreement is small, indicating acceptable agreement between the two methods except in specific circumstances mentioned below. The corresponding results for one-stage models are presented in Additional file 1.

**Fig. 3:**
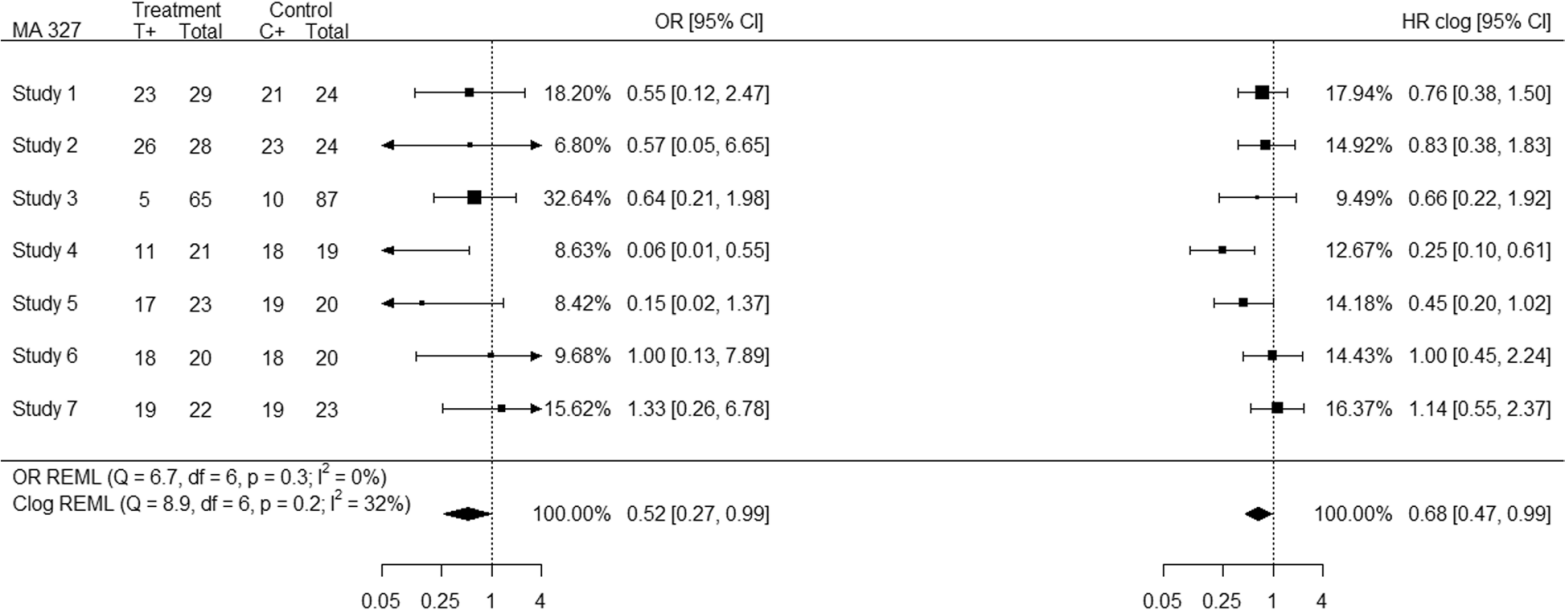
Forest plot (MA 327) indicating discrepancies in the presence of high event probability

Based on Bland–Altman plots, 6% (*n* = 47) of the meta-analyses were considered as outliers. In 21% of the “binary” outlying meta-analyses (e.g. MA 327; outlier obtained from ${I}^{2}$ estimates) a high event probability (defined here as probability greater than 0.7 for the majority of the individual studies) was observed. For example, meta-analysis 327 consists of 7 studies for which the event probability was greater than 0.7 for 5 out of 7 studies; consequently, high event probability affected substantially the differences in the individual study estimates between the OR and HR analyses, leading to different allocated relative weights for the studies, and discrepancies in the pooled effect estimates as shown in Fig. 3.

**Fig. 4:**
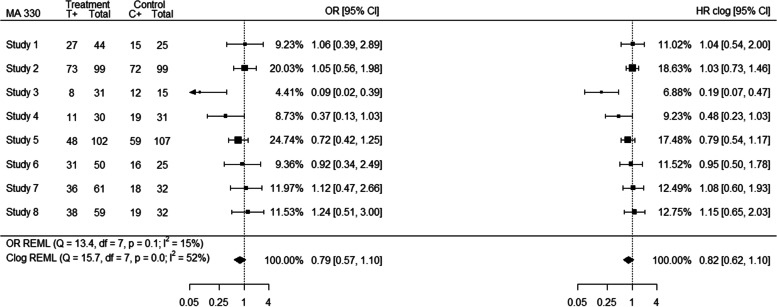
Forest plot (MA 330) indicating discrepancies arising from differences in between-study heterogeneity

The pooled HR estimates were closer to 1 than the OR estimates in the majority of meta-analyses (Additional file 1; outlier obtained from standardised and ${I}^{2}$ estimates) with the exception of MA 574 for “binary” data where, even though most of the individual study HR estimates are closer to 1 than the individual OR estimates, the pooled HR estimate is further from 1 than the pooled OR estimate. Increased within-study variability on the OR scale relative to the HR scale may affect the weighting more than the actual estimates in the studies, for example within “binary” data meta-analysis 7 (Additional file 1; outlier obtained from standardised estimates), producing some differences in the pooled effect estimates between the two scales. Important differences in between-study heterogeneity between the HR and OR analyses were also observed. For example, meta-analysis 330 (outlier obtained from ${I}^{2}$ estimates) consists of 8 studies of which 6 are smaller studies which received increased weight in the HR analysis compared to the OR analysis while the two larger studies received smaller weights; this affected both the individual HR estimates that have moved closer to each other and the relevant weights of the studies as presented in Fig. 4.

In 34% of the outlying meta-analyses, the individual study estimates and the corresponding weights were affected by a combination of differing event probability across study arms, differences in between-study heterogeneity or increased within-study variability on the OR relative to the HR scale. In the presence of a limited amount of studies in the meta-analyses this was even more evident. Additional examples of forest plots indicating the discrepancies among the results are shown in Additional file 1.

### Results for “OEV” data

In the Cochrane database, 157 meta-analyses were originally analysed using the “O-E” and “V” statistics on a HR scale. The median number of meta-analyses per review was 2 with IQR (2, 3). We observed that analysing time-to-event outcomes as HRs is restricted to very few medical specialties (Tables 2). For the “OEV” data, “Cancer” was still the most frequent medical specialty for both outcomes as observed in “binary” data (Table 2).

Table 3 provides the percentages of significant and non-significant meta-analyses for each outcome for two-stage models, indicating that discrepancies are more prevalent in the “OEV” data compared to the “binary” data; additionally the amount of discrepancies observed in statistical significance from the comparison of OR and HR obtained from the clog-log link was smaller than the amount of discrepancies observed between the OR and HR analyses.

**Fig. 5:**
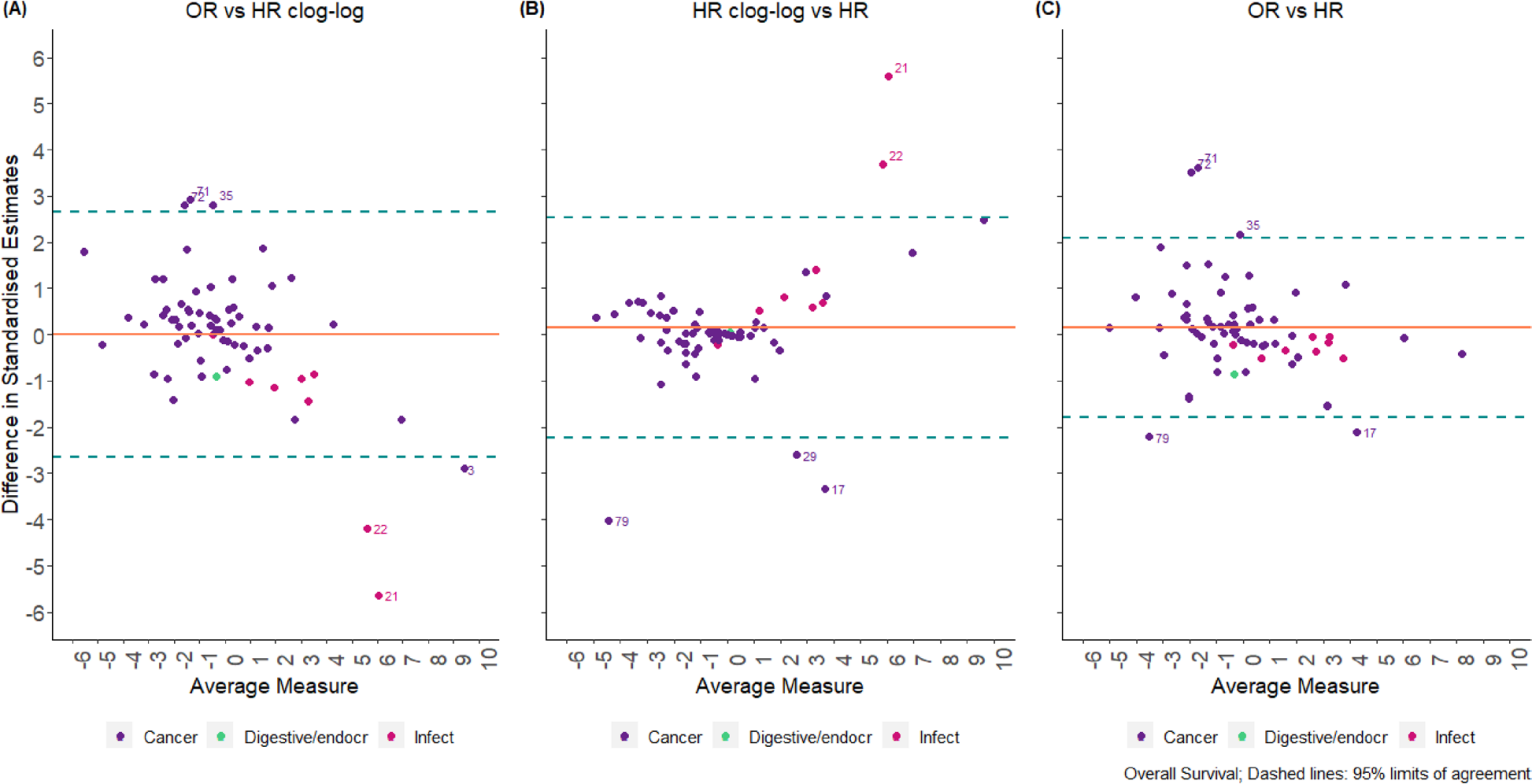
Bland–Altman Plot comparing standardised OR vs. HR estimates for two-stage models in “OEV” data

**Fig. 6:**
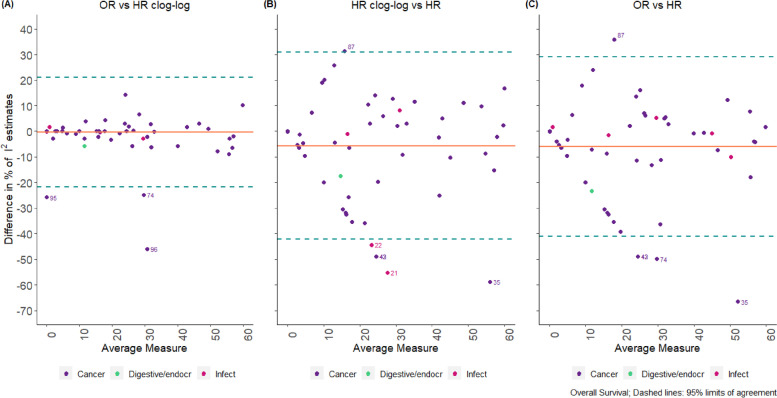
Bland–Altman Plot comparing ${I}^{2}$ estimates (OR vs. HR) for two-stage models in “OEV” data

Bland–Altman plots produced for “OEV” data indicated that the average difference between each pair of methods is larger than those obtained from the “binary” data (Figs. 5 and 6). For example, for overall survival, the average difference between the two methods for the standardised pooled effect estimates was 0.2 units (-1.8 units, 2.1 units) for OR versus HR and 0.2 units (-2.2 units, 2.5 units) for HR using clog-log versus HR; however, for OR vs HR clog-log differences the average bias was 0 units (-2.6 units, 2.7 units) indicating that clog-log is a closer approximation to OR rather than HR analyses (Fig. 5). For the estimation of I^2^, the average difference between the methods is -6% (-41%, 29%) for OR versus HR, -6% (-42%, 31%) for HR using clog-log versus HR, and 0% (-21%, 21%) for OR vs HR clog-log differences; similarly the clog-log seems a closer approximation to OR analyses rather than HR analyses (Fig. 6). The corresponding results for the outcome of progression/disease free survival are shown in Additional file 1.

**Fig. 7:**
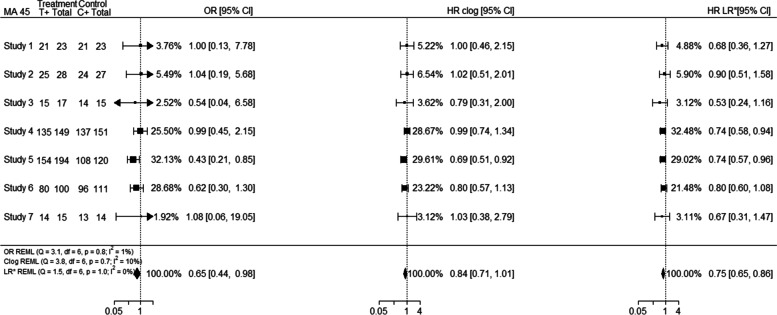
Forest plot (MA 45) indicating discrepancies in the presence of high event probability

Outliers were considered 28% of the “OEV” meta-analyses. Of these, 57% were from IPD rather than non-IPD and 54% of them were for the outcome of overall survival. In 50% of the outliers a high event probability (defined here as probability greater than 0.7) was observed, suggesting that this may be an important factor associated with differences among the scales used. For example, meta-analysis 45 (outlier obtained from standardised estimates) consists of 7 studies for which the event probability was greater than 0.7 for all the studies; consequently high event probability affected substantially the differences in the individual study estimates between the OR and HR analyses, leading to different allocated relative weights for the studies, and discrepancies in the pooled effect estimates as shown in Fig. 7. Even though the individual HR clog-log estimates were closer to the individual OR estimates the final pooled effect estimate was closer to the pooled HR estimate; this was not though the case for all meta-analyses.

**Fig. 8:**
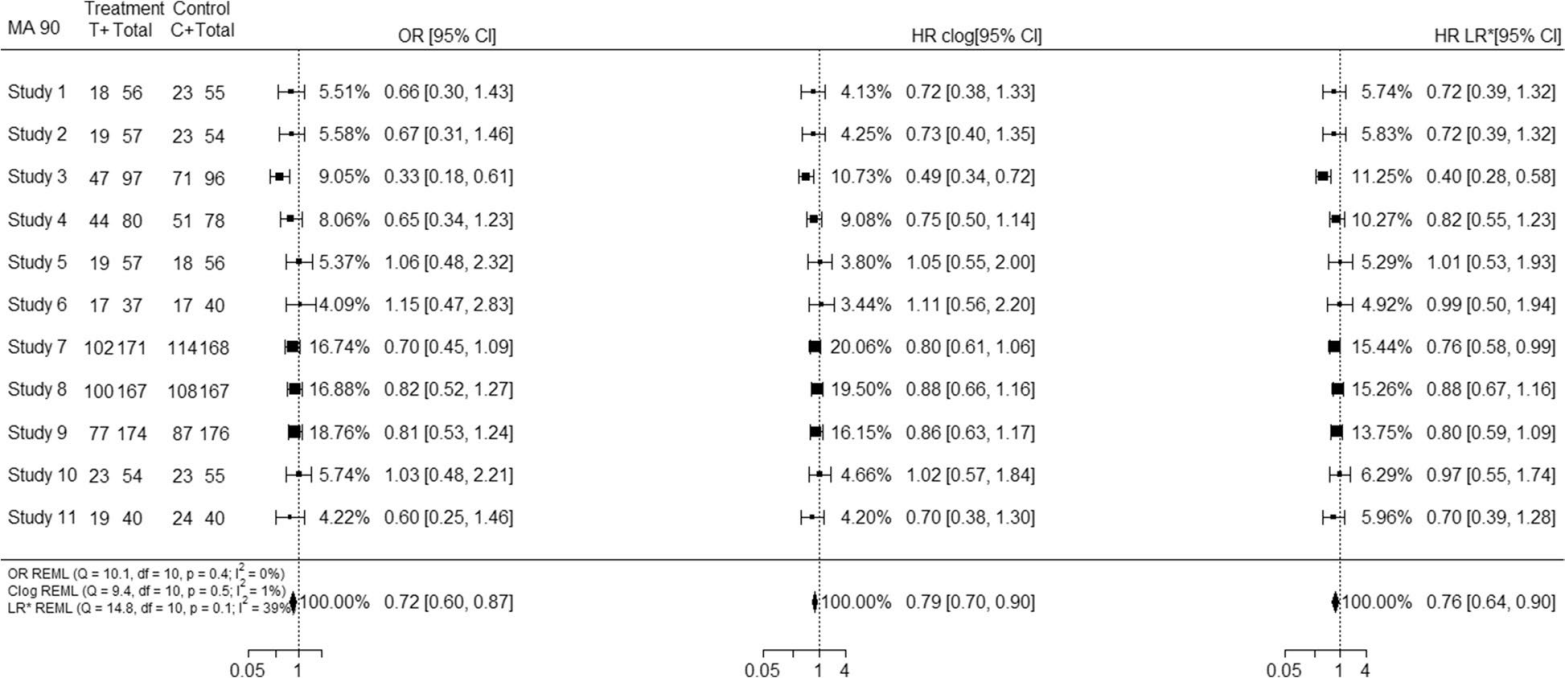
Forest plot (MA 90) indicating discrepancies arising from differences in between-study heterogeneity

Increased within-study variability on the OR scale relative to the HR scale may affect the weighting more than the actual estimates in the studies, for example for meta-analysis 17 (Additional file 1; outlier obtained from standardised estimates), producing differences in the pooled effect estimates between the two scales. Similarly, even though the individual study estimates and weights of OR and HR clog-log were closer to each other, the HR clog-log pooled effect estimate was closer to the pooled HR estimate; however, this was not the case for all meta-analyses. Important differences in between-study heterogeneity between the HR and OR analyses were observed in meta-analyses such as 42, 90. For example, meta-analysis 90 (outlier obtained from ${I}^{2}$ estimates) consists of 11 studies out of which 8 are smaller studies and 3 are larger studies. Smaller studies received increased weight in the HR analysis compared to the OR analysis, while larger studies received smaller weights in the HR scale compared to OR scale. However, this was not the case on the HR clog-log scale as presented in Fig. 8.

In 46% of the outlying meta-analyses, the individual study estimates, and the corresponding weights were affected by a combination of differing event probability across study arms, differences in between-study heterogeneity or increased within-study variability on the OR relative to the HR scale. In the presence of a limited amount of studies in the meta-analyses this was even more evident. Additional forest plots indicating the discrepancies among the results are shown in Additional file 1.

Overall, using the “OEV” data, a mixed pattern was observed. In 39% (*n* = 11) of outlying meta-analyses the OR pooled effect estimate was closer to HR pooled effect estimate; however in 4 out of 11 outlying meta-analyses the individual study estimates obtained from the HR clog-log link were a closer approximation to the individual study HR estimates. Similarly, even though in 61% (*n* = 17) of the outlying meta-analyses the HR clog-log pooled effect estimate was closer to the pooled HR estimate, 3 of outlying meta-analyses provided individual study OR estimates closer to individual study HR estimates, and another 3 individual study HR clog-log estimates were closer to individual study OR estimates.

## Discussion

Using meta-analysis data from the CDSR of 2008, we investigated how time-to-event outcomes are treated within meta-analysis; we explored the differences that occur when data are analysed as binary as opposed to analysing the data using the complementary log–log link or using the “O-E” and “V” statistics where interpretation is conducted on a HR scale. For both datasets, we identified important reasons associated with discordance among the results, indicating that the correct choice of the method does matter and may affect the interpretation and conclusions drawn from the results. Our analyses highlighted that high event probability was an important factor associated with discordant effect estimates; changes to between and within-study variation were important mechanisms producing differences in the results as well. However, there were occasions where there was no clear single factor driving the differences, since there was a combination of reasons affecting the individual study estimates and corresponding weights. Regarding method selection, based on the “OEV” data we identified that a mixed pattern was observed and there was no clear indication under which exact conditions the clog-log link outperforms logit link on an OR scale and vice versa.

While most of the meta-analyses within the database were analysed originally as binary, with an outcome classification of all-cause mortality it is worth mentioning that these meta-analyses could include the outcome of short-term mortality (e.g. 30 days) or longer-term mortality (e.g. 5 years); therefore some of these meta-analyses with short follow-up may have been appropriately analysed as binary. The outcome classification of all-cause mortality was considered a representative sample of survival meta-analysis up to 2008, however results might be different for other outcomes and results might have changed in later reviews where more information on methodology was available. The data used for the comparison of OR/HR scale in the “OEV” data were slightly different; we used the number of events and non-events for the OR and HR clog-log calculation (as in “binary” data) and calculated a HR based on “O-E” and V statistics. Therefore, there is a possibility for some cases that the two data sets entered by Cochrane reviewers may not completely correspond to each other.

We did not assess other reasons for differences between the results due to lack of information on censoring and follow-up times. Interpretation of the results was conducted with caution as we are interpreting the results based on known factors, without excluding other unknown factors that may have affected the results. We were not able to examine whether current practice of analysing time-to-event data has changed and whether methodological choices have improved since 2008. Further work examining the differences observed between analyses on the OR and HR scales in the presence of IPD is necessary.

The model used to analyse time-to-event data as binary is the conventional approach widely used by many systematic reviewers and meta-analysts [19]. It is quick, inexpensive and study results are obtained from appropriately synthesized study publications or by contacting study authors [20]. This approach to analysis ignores censored observations [21] and treats them as missing and has also been criticised for the within-study normality assumptions required [20].

The use of a clog-log link function, facilitating the results’ interpretation in a HR scale for both “binary” and “OEV” data, was the best alternative approach enabling us to make comparisons between the scales used if only binary summaries are available. In the past, the clog-log link has been proven to provide a close approximation to Cox regression invoking a proportional hazards assumption, rather than a proportional odds assumption [6]. However, due to lack on information on “O-E” and “V” statistics for “binary” data only, we were not able to assess whether the HR obtained from the clog-log link is a close approximation to the true HR; therefore this magnifies the importance of extracting appropriate information when conducting time-to-event meta-analysis. For the “OEV” data, “O-E and V” data provide the best method to analyse aggregate data and facilitate results’ interpretation on the HR scale but in the absence of IPD important biases may occur when large treatment effects and unbalanced data are present [22]. Additionally, we were not able to identify a clear pattern under which the complementary log–log link could be employed since there were circumstances under which it performed better or worse than an OR analysis; therefore we were not able to identify whether the clog-log approach is useful when a MA includes binary summaries alongside OEV or HR summaries. IPD and simulation studies are required to assess in more detail the conditions determining where this method would be acceptable.

For the “binary” data, we also used a one-stage random-effects model with fixed study-specific effects describing the baseline risk probability of the event in each study. These models use exact binomial likelihoods and may therefore be more accurate, especially with sparse data [14]. The fixed study-specific effects cause difficulties in estimation since the number of parameters increases with the number of studies, but maximum likelihood theory requires the number of parameters to remain stable as the sample size increases. A random-effects model with random study-specific effects could be applied, however based on simulation studies this model performed better than others without any serious biases present [14]. We were not able to make comparisons using one-stage models in the “OEV” data. We would be able to apply one-stage models when the data were analysed as binary, but we did not have the IPD required to fit one-stage models on the HR scale.

To our knowledge, no research has been conducted using such a large database assessing the differences between a) analysing the data as binary and interpreting the results in an OR scale and b) analysing the data either using the clog-log link or log-rank “O-E” and V statistics facilitating interpretation on the HR scale.

We have demonstrated the impact of reanalysing meta-analyses (“binary” or “OEV” datasets) within the Cochrane Database on a different scale, identifying the main drivers influencing discrepancies between the meta-analytic results. Our findings provide useful insights into changes to meta-analytical results and indicate that choice of method used in meta-analysis of survival data does matter, especially in the presence of high event probabilities.

## Conclusions

In conclusion, our findings indicate that time-to-event data should be ideally analysed accounting for their natural properties, as it is possible for important discrepancies to be observed and conclusions from the meta-analysis to be altered. We identified that dichotomising time-to-event outcomes may be adequate for low event probabilities but not for high event probabilities. In meta-analyses where only binary data are available, the complementary log–log link may be a useful alternative when analysing time-to-event outcomes as binary, however the exact conditions need further exploration. These findings provide guidance on the appropriate methodology that should be used when conducting such meta-analyses.

## Supplementary Information

**Additional file 1: Section 1. **Fitting one-stage random-effects models for “binary” data. **Section 2. **Number (%) of (non-)significant meta-analyses under different scales for one-stage models (“binary” data). **Section 3. **Bland-Altman plots comparing standardised pooled effect and ${I}^{2}$ estimates for one-stage models (“binary” data). **Section 4.** Forest plots for example MAs considered as outliers in our analyses (“binary” data). **Section 5. **Bland-Altman Plot comparing standardised OR vs. HR estimates for two-stage models in “OEV” data. **Section 6. **Forest plot for example MAs considered as outliers in our analyses (“OEV” data). Section 7: R Code

## Data Availability

Data are available upon reasonable request, if permission is obtained from Cochrane.

## Abbreviations

CDSR: Cochrane Database of Systematic Reviews
HR(s): Hazard Ratio(s)
IQR: Interquartile Range
IPD: Individual Participant Data
MA(s): Meta-analysis(es)
OEV: Observed minus Expected and Variance statistics
OR(s): Odds Ratio(s)
REML: Restricted Maximum Likelihood
RR(s): Risk Ratio(s) or Relative Risk(s)
SR(s): Systematic Review(s)
