# Supplementary material for: Implications of analysing time-to-event outcomes as binary in meta-analysis: empirical evidence from the Cochrane Database of Systematic Reviews
Source: BMC Med Res Methodol. 2022 Mar 20;22:73. doi: 10.1186/s12874-022-01541-9 (PMC8934481; doi:10.1186/s12874-022-01541-9)
Supplement: Supplementary file 1 — Additional file 1: Section 1. Fitting one-stage random-effects models for “binary” data. Section 2. Number (%) of (non-)significant meta-analyses under different scales for one-stage models (“binary” data). Section 3. Bland-Altman plots comparing standardised pooled effect and ${I}^{2}$I2 estimates for one-stage models (“binary” data). Section 4. Forest plots for example MAs considered as outliers in our analyses (“binary” data). Section 5. Bland-Altman Plot comparing standardised OR vs. HR estimates for two-stage models in “OEV” data. Section 6. Forest plot for example MAs considered as outliers in our analyses (“OEV” data). Section 7: R Code [file 12874_2022_1541_MOESM1_ESM.docx]

**Additional File 1**

## Section 1: Fitting one-stage random-effects models for “binary” data

Let $i=1,2,\ldots,n$ denote the study and $k=0, 1$ denote the treatment group ($k=0$ indicates control and $k=1$ indicates active treatment group). We assume that $\pi_{ik}$ is the event probability in the $i^{th}$ study for the $k^{th}$ treatment group.

A generalised linear mixed model was fitted first from Jackson et al.^14^ for the “binary” data and we extended it to the HR scale. This model uses the exact binomial likelihood allowing us to provide more accurate results, especially with sparse data^14^. According to the Simmonds and Higgins model^17^ we assumed that:

$g(\pi_{ik})=\gamma_{i}+k\cdot\theta_{i}$*,*$\theta_{i}\sim N(\theta,\tau^{2})$ (7)

where $g(\pi_{ik})$ is a link function with:

$$g(\pi_{ik})=\left\{ \begin{matrix} logit\left( \pi_{ik} \right) for ORs \\ log[-\log\left( 1-\pi_{ik} \right)] for HRs \end{matrix} \right.$$

$\gamma_{i}$ was the baseline risk of event in study $i$, $\theta$ was the overall treatment effect across studies, $\tau^{2}$ was the heterogeneity across studies, $\theta_{i}$ was the true study-specific treatment effect which varies between studies. Using the glmer function in R, we obtain the following:

$g(\pi_{ik})=\gamma_{i}+k\cdot\theta+k\cdot\varepsilon_{i}$, where $\varepsilon_{i}\sim N(0,\tau^{2})$ and all $\varepsilon_{i}$ are independent. We applied to our dataset a modification of the Simmonds and Higgins model with random treatment effects and fixed study-specific effects indicating that there is a separate baseline risk parameter $\gamma_{i}$ for each study as follows:

$g(\pi_{ik})=\gamma_{i}+k\cdot\theta+z_{ik}\varepsilon_{i}$ (8)

We replaced $k\cdot\theta$ from the above equation with $z_{ik}\theta=(k-0.5)\theta$. The model’s form does not change and $z_{ik}\theta$ is only a re-parameterisation of the model as described in detail by Jackson et al.^14^ The “rma.glmm” command from “metafor” package was used to calculate the one-stage ORs and the “glmer” command from “lme4” package was used for the corresponding HR estimates. Estimation of between-study heterogeneity ($I^{2}$) for the one-stage HR models was considered computationally intensive^18^ and was computed outside the model specification; to provide justification for our method of calculation, $I^{2}$estimates were obtained similarly for the one-stage OR models and were compared to the directly modelled ones, indicating almost identical results.

**Section 2:** Number (%) of (non-)significant meta-analyses under different scales for one-stage models (“binary” data).

|  |  |  | **OR** | |
| --- | --- | --- | --- | --- |
|  | Outcome |  | Significant | Non-significant |
| **HR** | All-cause mortality  (One-stage models) | Significant | 123 (17%) | 2 (0.3%) |
|  |  | Non-significant | 4 (0.6%) | 589 (82%) |

**Section 3:** Bland-Altman plots comparing standardised pooled effect and $I^{2}$ estimates for one-stage models (“binary” data).


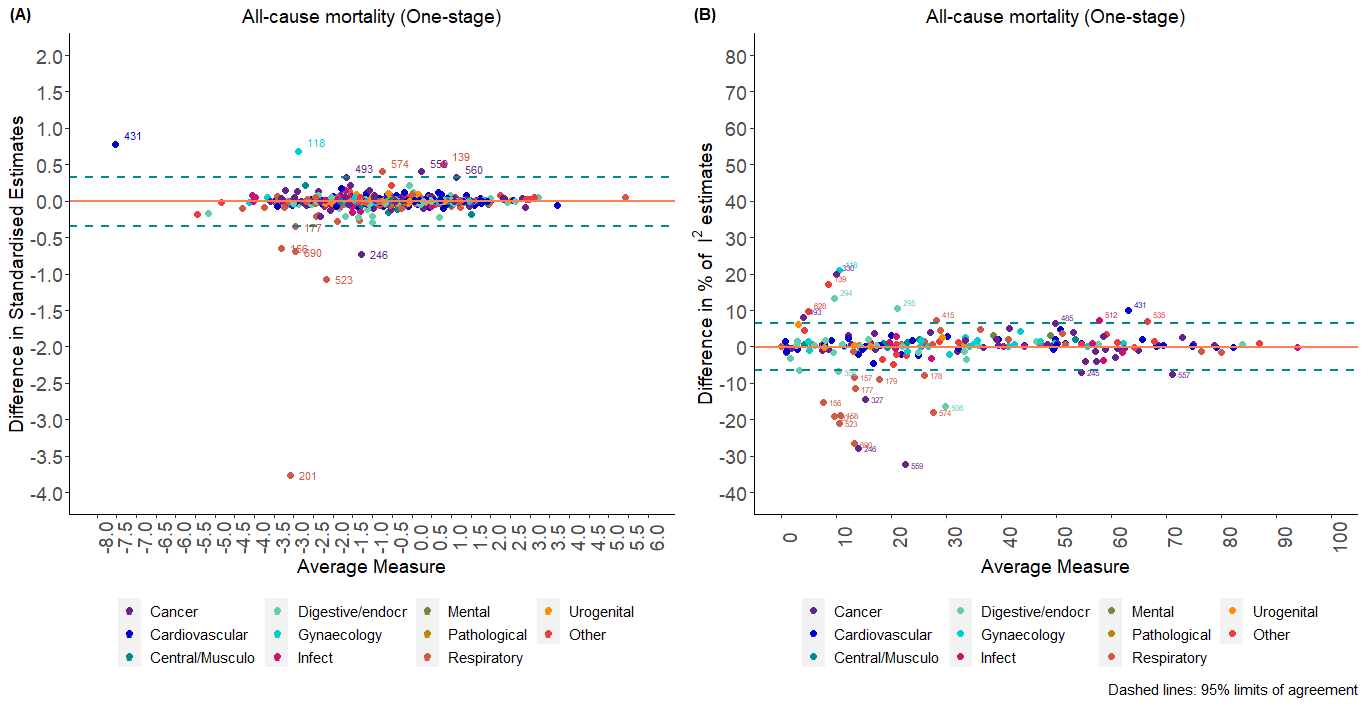


**Section 4:** Forest plots for example MAs considered as outliers in our analyses (“binary” data).

MA 574: Pooled OR estimate closer to one than pooled HR estimate.


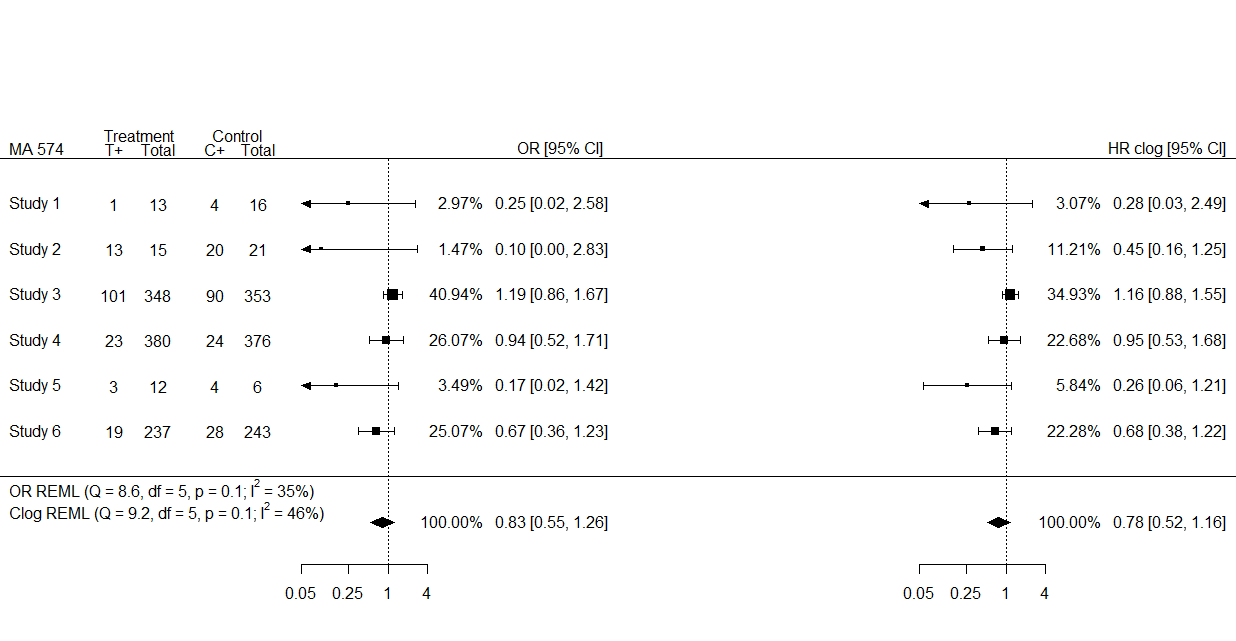


MA 7: Increased within-study variability on the OR scale relative to the HR scale


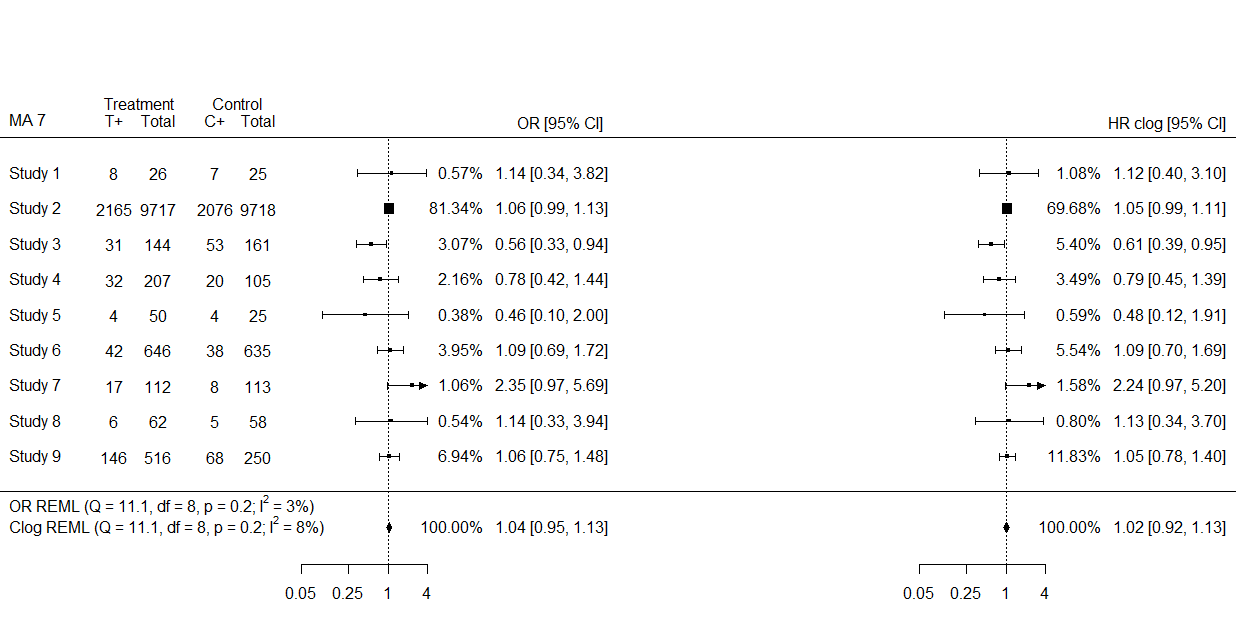


MA 525: Combination of reasons affecting the OR scale relative to the HR scale


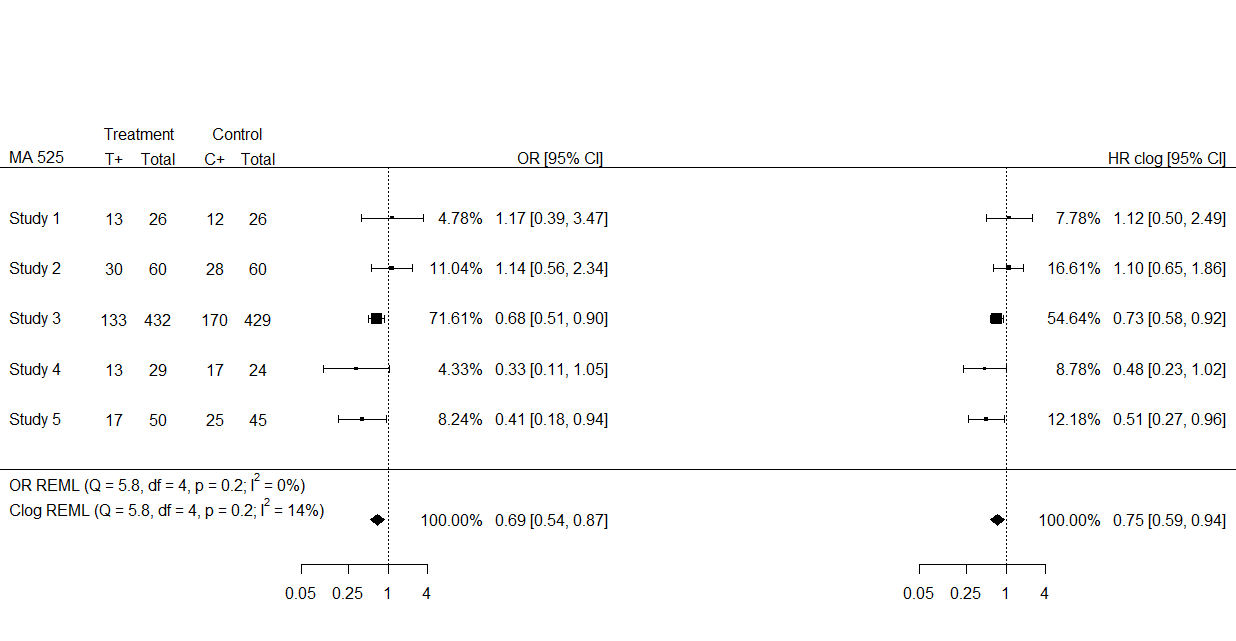


**Section 5:** Bland-Altman Plot comparing standardised OR vs. HR estimates for two-stage models in “OEV” data.


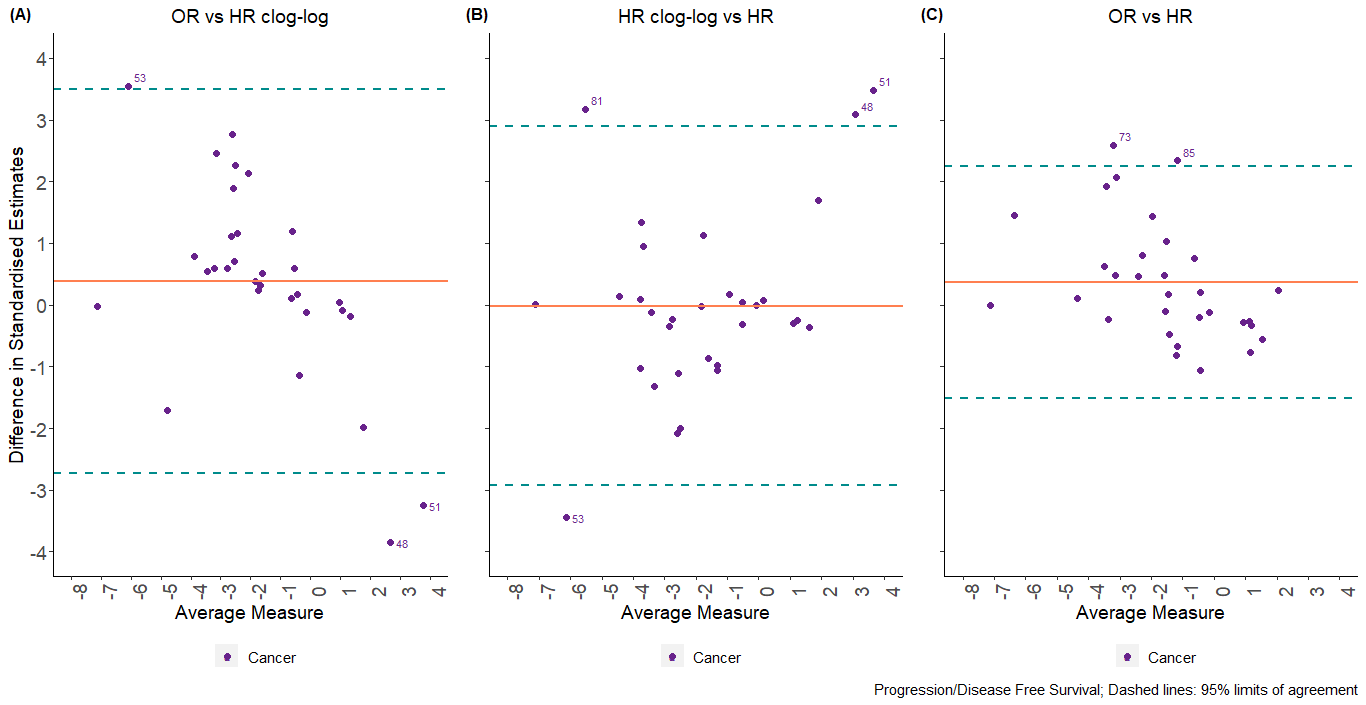


Bland-Altman Plot comparing $I^{2}$ estimates (OR vs. HR) for two-stage models in “OEV” data.


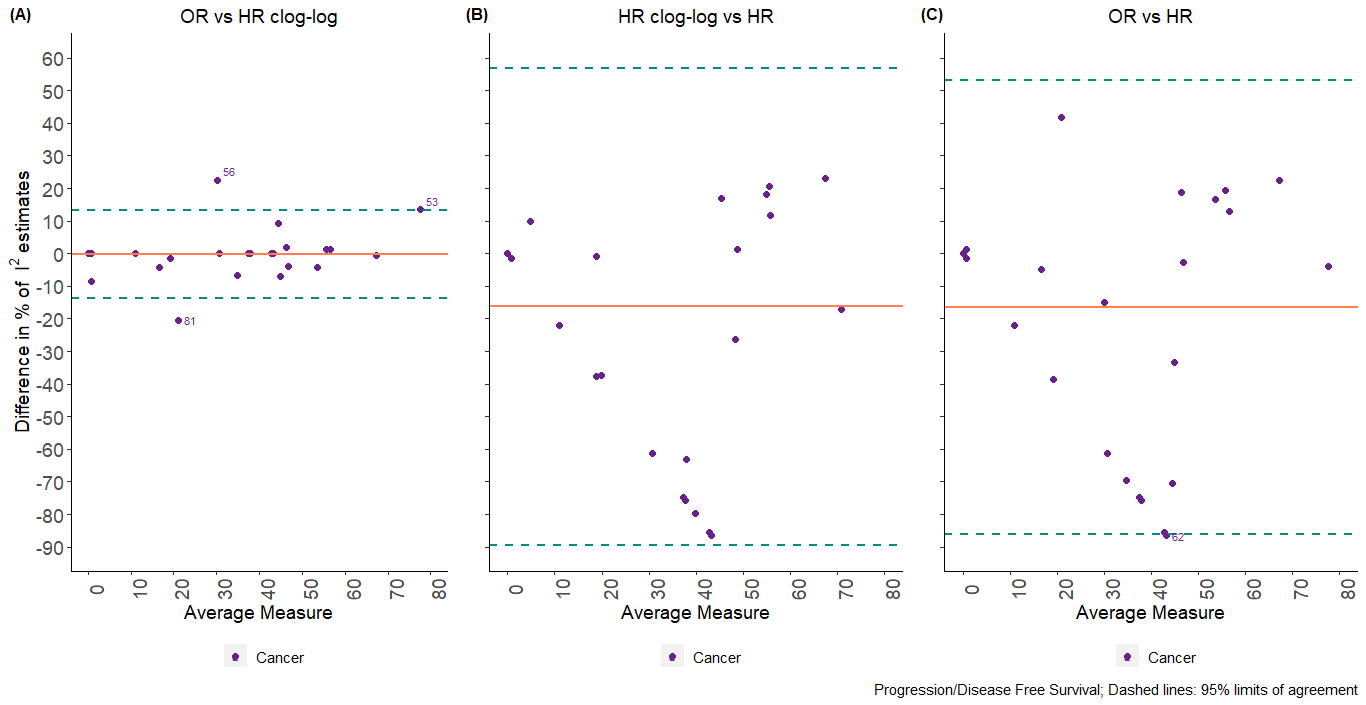


**Section 6:** Forest plot for example MAs considered as outliers in our analyses (“OEV” data).

MA17: Increased within-study variability on the OR scale relative to the HR scale.


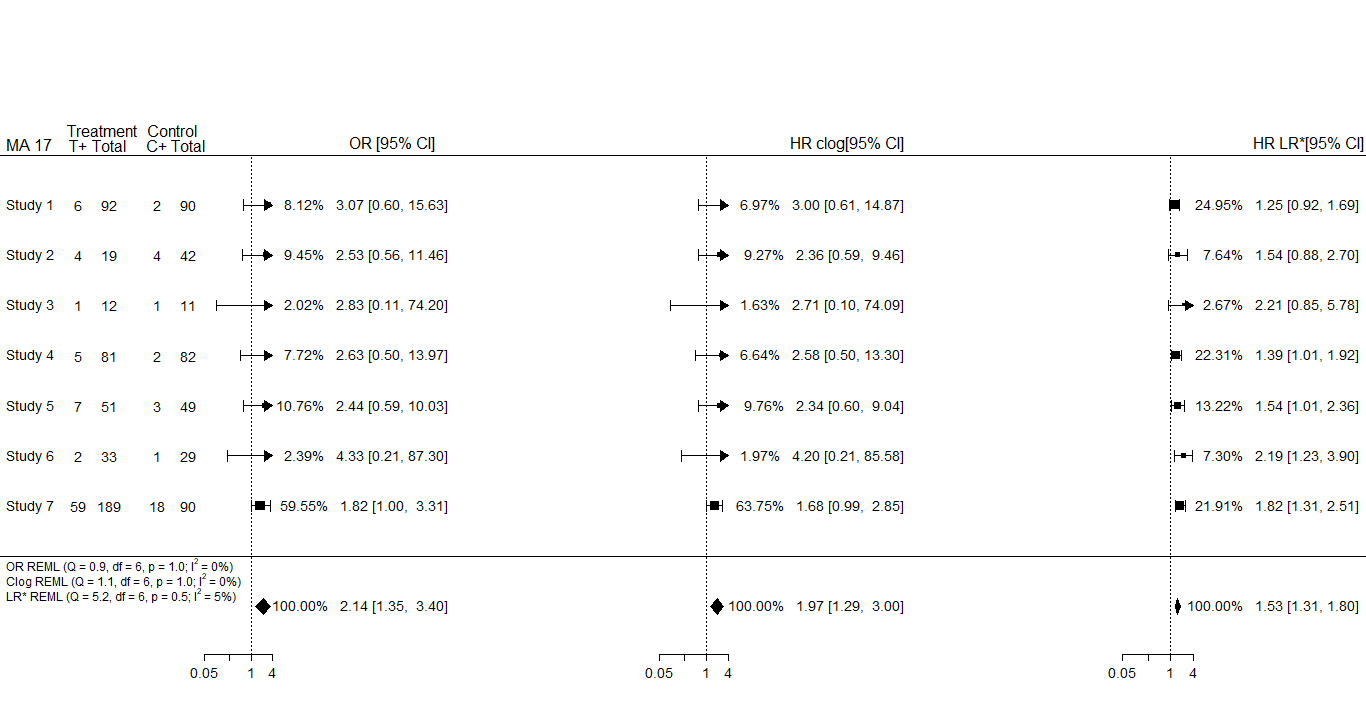


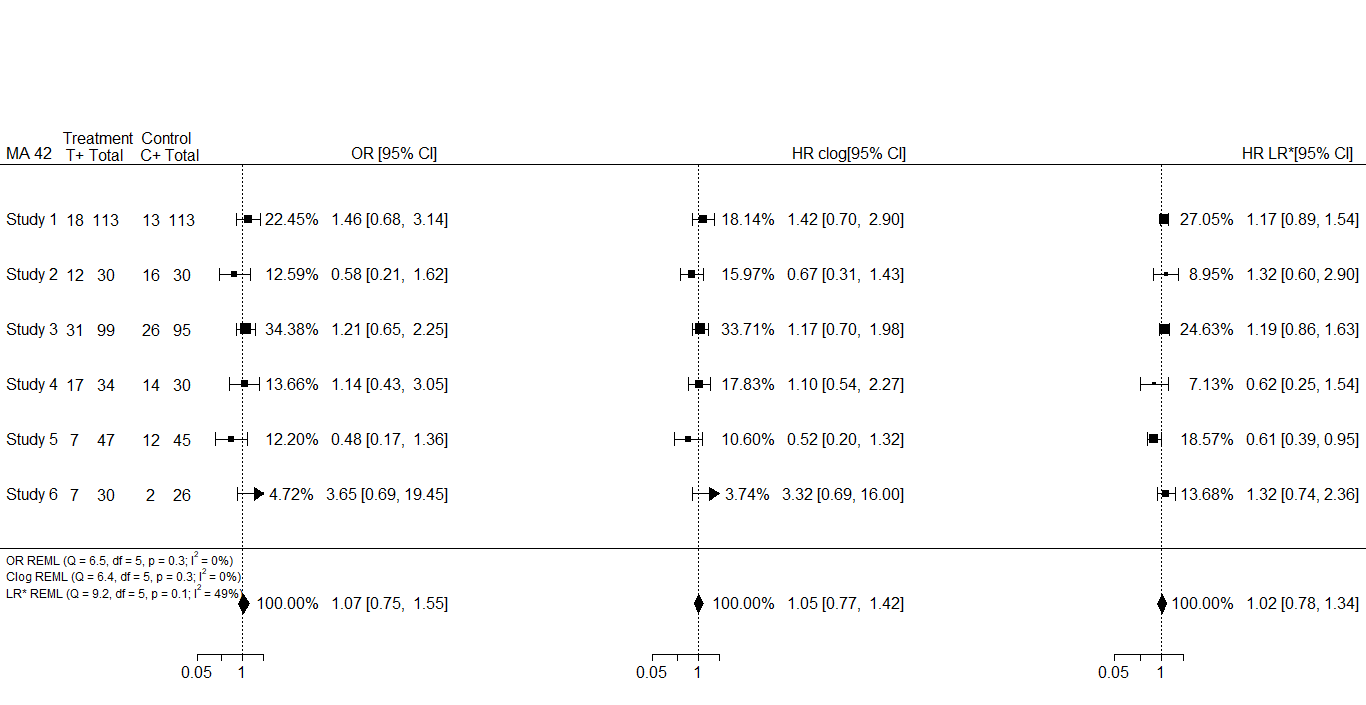


MA 42: Forest plot indicating discrepancies arising from differences in between-study heterogeneity.

MA 81: Combination of reasons affecting the OR scale relative to the HR scale


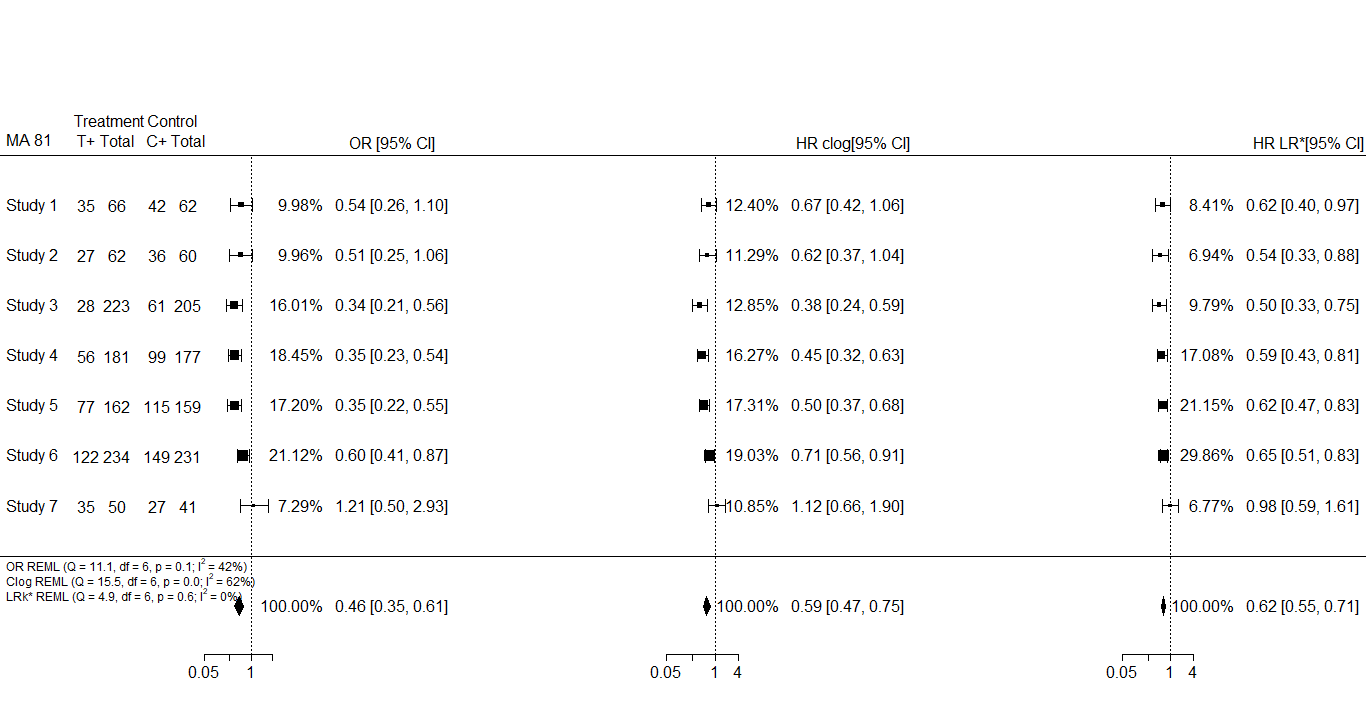


**Section 7: R** Code

Two-stage models for ORs

for (i in unique(CDSR_2008$ma)) {

cat(i,"\n")

try.fit<- try(rma.uni(ai = treat_n, bi = nontreat_n, ci = ctrl_n, di = nonctrl_n,

data = CDSR_2008[CDSR_2008$ma==i,], measure = "OR",method="REML",

control=list(maxiter=500, verbose=TRUE, stepadj=0.5), verbose=TRUE))

resultsREML[i,7]<-i

resultsREML[i,8]<-unique(CDSR_2008[CDSR_2008$ma==i,]$scode)

if (class(try.fit)!="try-error") {

CDSR.2008[[i]]<- try.fit

resultsREML[i,1]<-as.numeric(exp(CDSR.2008[[i]]$b))

resultsREML[i,2]<-as.numeric(CDSR.2008[[i]]$se)

resultsREML[i,3]<-as.numeric(exp(CDSR.2008[[i]]$ci.lb))

resultsREML[i,4]<-as.numeric(exp(CDSR.2008[[i]]$ci.ub))

resultsREML[i,5]<-as.numeric(CDSR.2008[[i]]$tau2)

resultsREML[i,6]<-as.numeric(CDSR.2008[[i]]$I2)

} else {

CDSR.2008[[i]] <- NULL }}

Two-stage models for HRs using the clog-log link or “O-E” and “V” statistics

for (i in unique(CDSR_2008$ma)) {

cat(i,"\n")

try.fit1<- try(rma.uni(yi = logHR, vi = varHR, data = CDSR_2008[CDSR_2008$ma==i,],

method="REML",control=list(maxiter=10e9, verbose=TRUE, stepadj=0.2), verbose=TRUE))

# Try the following line if you want to obtain HR estimates based on “O-E” and “V” statistics. Ignore otherwise

# try.fit1<- try(rma.uni(yi = logHR, vi = varHR, data = CDSR_2008[CDSR_2008$ma==i,],

# method="REML",control=list(maxiter=10e9, verbose=TRUE, stepadj=0.2), verbose=TRUE))

resultsREMLHR[i,7]<-i

resultsREMLHR[i,8]<-unique(CDSR_2008[CDSR_2008$ma==i,]$scode)

if (class(try.fit1)!="try-error") {

CDSR.2008HR[[i]]<- try.fit1

resultsREMLHR[i,1]<-as.numeric(exp(CDSR.2008HR[[i]]$b))

resultsREMLHR[i,2]<-as.numeric(CDSR.2008HR[[i]]$se)

resultsREMLHR[i,3]<-as.numeric(exp(CDSR.2008HR[[i]]$ci.lb))

resultsREMLHR[i,4]<-as.numeric(exp(CDSR.2008HR[[i]]$ci.ub))

resultsREMLHR[i,5]<-as.numeric(CDSR.2008HR[[i]]$tau2)

resultsREMLHR[i,6]<-as.numeric(CDSR.2008HR[[i]]$I2)

} else {

CDSR.2008HR[[i]] <- NULL }}

One-stage MA for ORs

resultsUMFS=data.frame(matrix(NA, max(CDSR_2008$ma), 8))

colnames(resultsUMFS)<-c("estimates","SE", "LowerCI", "UpperCI","Tau", "Isq", "MA", "Med_Area")

for (i in unique(CDSR_2008$ma)) {

cat(i,"\n")

try.fit2<- try(rma.glmm(ai = treat_n, bi = nontreat_n, ci = ctrl_n, di = nonctrl_n,

data = CDSR_2008[CDSR_2008$ma==i,], measure = "OR",model="UM.FS", drop00=F,nAGQ=7))

resultsUMFS[i,7]<-i

resultsUMFS[i,8]<-unique(CDSR_2008[CDSR_2008$ma==i,]$scode)

if (class(try.fit2)!="try-error") {

CDSR.2008stg1[[i]]<- try.fit2

resultsUMFS[i,1]<-as.numeric(exp(CDSR.2008stg1[[i]]$b))

resultsUMFS[i,2]<-as.numeric(CDSR.2008stg1[[i]]$se)

resultsUMFS[i,3]<-as.numeric(exp(CDSR.2008stg1[[i]]$ci.lb))

resultsUMFS[i,4]<-as.numeric(exp(CDSR.2008stg1[[i]]$ci.ub))

resultsUMFS[i,5]<-as.numeric(CDSR.2008stg1[[i]]$tau2)

resultsUMFS[i,6]<-as.numeric(CDSR.2008stg1[[i]]$I2)

} else {

CDSR.2008stg1[[i]] <- NULL}}

One-stage MA for HRs

resultsUMFSHR=data.frame(matrix(NA, max(datlong.CDSR_2008$ma.num), 7))

colnames(resultsUMFSHR)<-c("estimates","SE", "Tau", "LowerCI", "UpperCI", "MA", "Med_Area")

for (i in unique(datlong.CDSR_2008$ma.num)) {

cat(i,"\n")

try.fit3<-try(glmer(cbind(event,n-event) ~ factor(treat) + factor(study) + (treat12-1|study),

data=datlong.CDSR_2008[datlong.CDSR_2008$ma.num==i,], family=binomial(link="cloglog"),nAGQ=7, drop00=F))

resultsUMFSHR[i,6]<-i

resultsUMFSHR[i,7]<-unique(datlong.CDSR_2008[datlong.CDSR_2008$ma.num==i,]$medical.area)

if (class(try.fit3)!="try-error") {

CDSR.2008long[[i]]<- try.fit3

CDSR.2008long1.CI[[i]]<-confint.merMod(CDSR.2008long[[i]], method="Wald")

resultsUMFSHR[i,1]<-as.numeric(exp(summary(CDSR.2008long[[i]])$coeff[2,1]))

resultsUMFSHR[i,2]<-as.numeric(summary(CDSR.2008long[[i]])$coeff[2,2])

resultsUMFSHR[i,3]<-as.numeric(summary(CDSR.2008long[[i]])$varcor)

resultsUMFSHR[i,4]<-exp(CDSR.2008long1.CI[[i]][3,1])

resultsUMFSHR[i,5]<-exp(CDSR.2008long1.CI[[i]][3,2])

} else {

CDSR.2008long[[i]] <- NULL}}
